# Supplementary material for: Resolving the structural basis of therapeutic antibody function in cancer immunotherapy with RESI
Source: Nat Commun. 2025 Jul 23;16:6768. doi: 10.1038/s41467-025-61893-w (PMC12284157; doi:10.1038/s41467-025-61893-w)
Supplement: Supplementary file 1 — Supplementary Information [file 41467_2025_61893_MOESM1_ESM.pdf]

Supplementary Information

Resolving the structural basis of therapeutic antibody function in cancer immunotherapy with RESI

Isabelle Pachmayr[1,2], Luciano A. Masullo[1], Susanne C.M. Reinhardt[1,3], Jisoo Kwon[1], Maite Llop[4], Ondřej Skořepa[1,5], Sylvia Herter[4], Marina Bacac[4], Christian Klein[2,4], Ralf Jungmann[1,3,*]

[1]Max Planck Institute of Biochemistry, Planegg, Germany, [2]Department of Biochemistry, Ludwig Maximilian University, Munich, Germany, [3]Faculty of Physics and Center for Nanoscience, Ludwig Maximilian University, Munich, Germany, [4]Roche Innovation Center Zurich, Roche Pharma and Early Development, Schlieren, Switzerland, [5]Department of Biochemistry, Charles University, Prague, Czech Republic. Correspondence should be addressed to R.J. (jungmann@biochem.mpg.de)

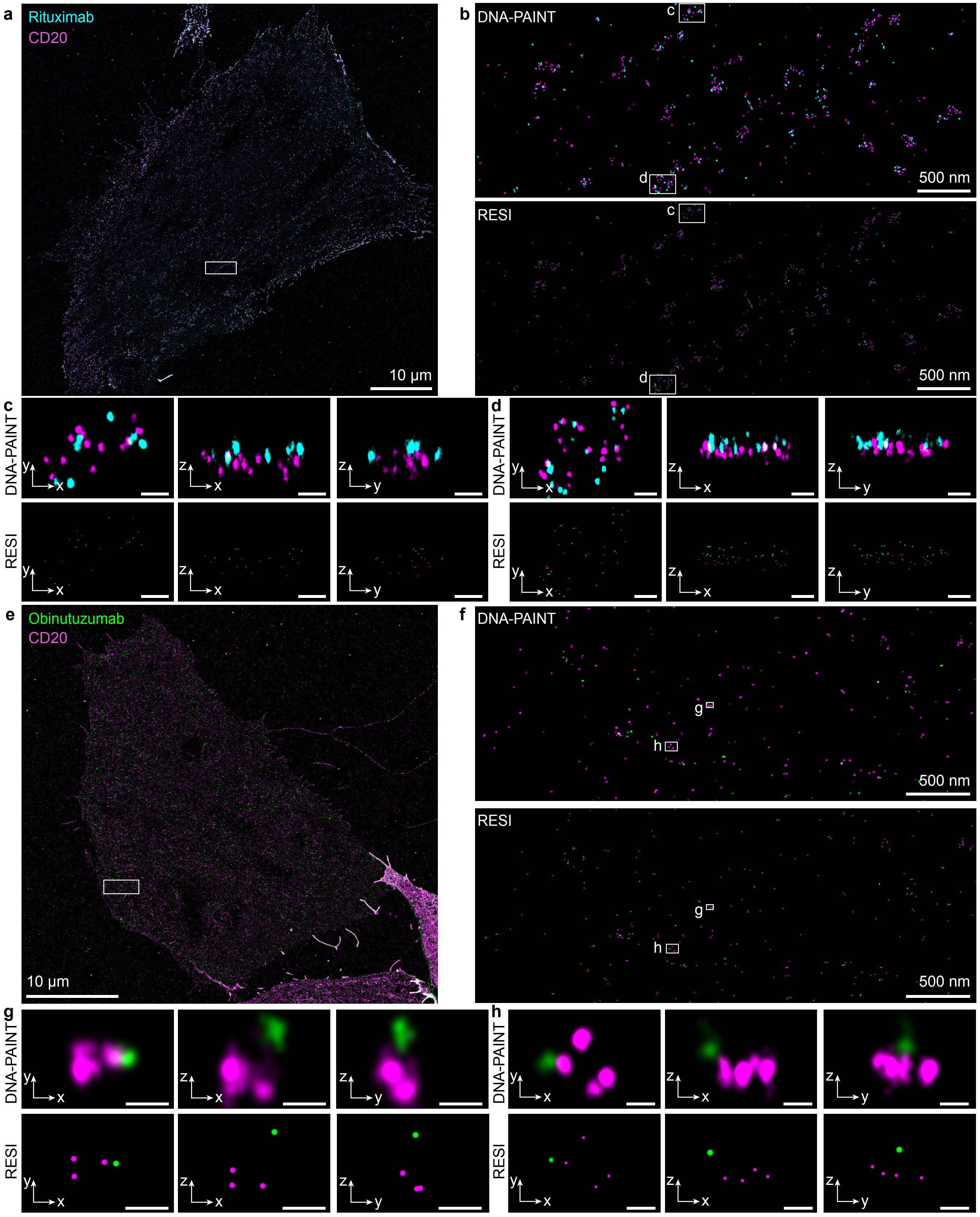

a
Rituximab
CD20
10 µm
b
DNA-PAINT
RESI
500 nm
c
d
DNA-PAINT
RESI
e
Obinutuzumab
CD20
10 µm
f
DNA-PAINT
RESI
g
h
500 nm

Supplementary Fig. 1 | Replicate for super-resolution imaging of CD20-therapeutic antibody complexes using RESI. **a**, DNA-PAINT imaging shows that Rituximab (RTX) (cyan) colocalizes with CD20 (magenta), forming distinct higher-order structures. **b**, RTX-CD20 complexes exhibit clustered formations. RESI resolution allows visualization of individual proteins. **c** and **d**, 3D view of individual RTX-CD20 complexes. **e**, DNA-PAINT imaging shows that Obinutuzumab (OBZ) (green) and CD20 (magenta), are homogeneously distributed in the cell. **f**, Transitioning from DNA-PAINT super-resolution to RESI resolution allows visualization of OBZ-CD20 complexes without evident higher-order clustering. **g** and **h**, 3D view of individual OBZ-CD20 complexes, lacking the distinct planar higher-order structures observed in RTX-treated cells. Scale bars in (**c**, **d**): 50 nm. Scale bars in (**g**, **h**): 20 nm.

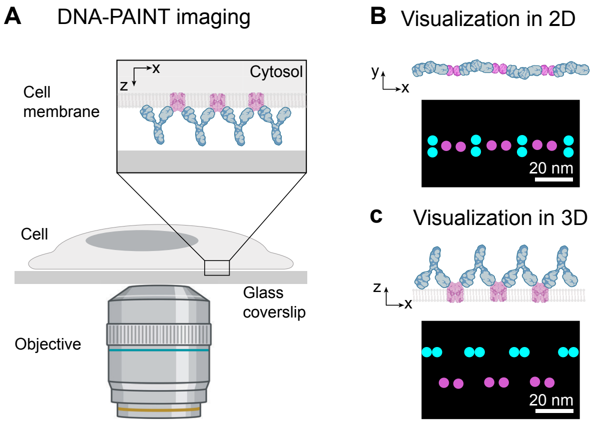

Supplementary Fig. 2 | Visualization of mAb-CD20 complexes in adherent cells. a, DNA-PAINT imaging with an inverted microscope. **b,** For 2D visualization, images are displayed in the x-y-plane. **c,** For 3D visualization, images are rotated around the x-axis. Created with the help of BioRender (https://BioRender.com/y76v9f4 and https://BioRender.com/5575210)

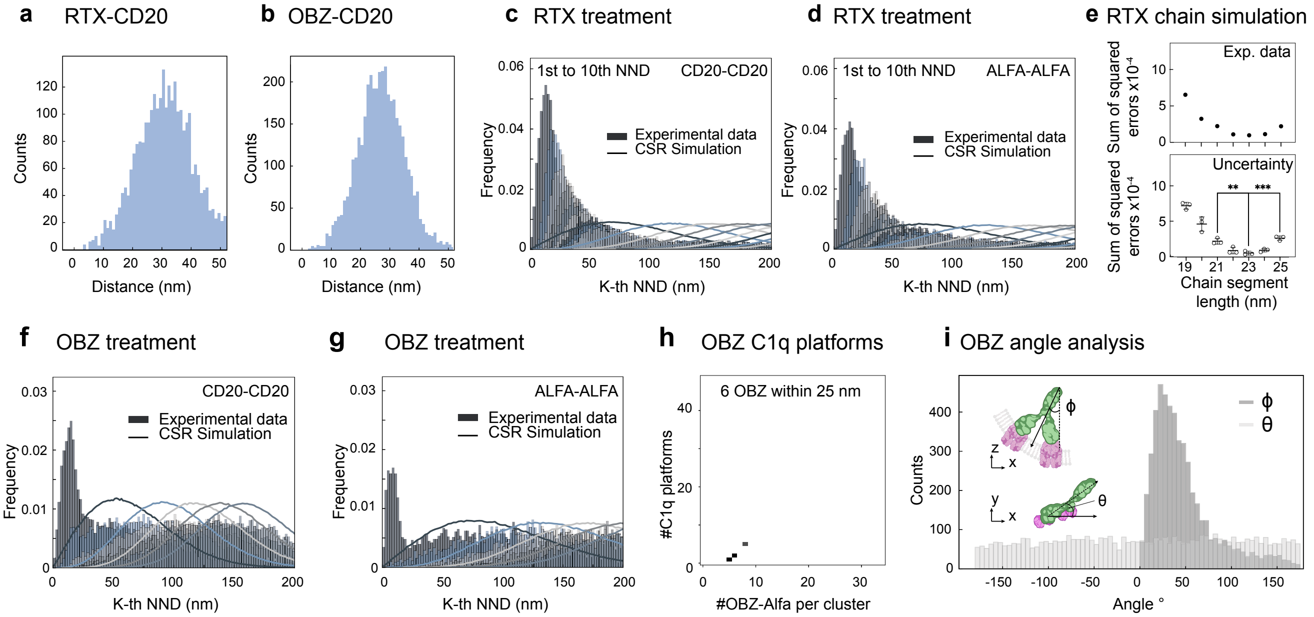

Supplementary Fig. 3 | Quantitative analysis of therapeutic Antibody-CD20 complexes. a, Axial RTX to CD20 distances within RTX-CD20 complexes are 32 nm ± 11 nm (mean ± std). **b,** Axial OBZ to CD20 distances within OBZ-CD20 complexes are 27 nm ± 9 nm (mean ± std). **c,** Nearest-Neighbor Distance (NND) analysis of CD20 after RTX treatment shows non-CSR distributions for all shown NNDs. **d,** Nearest-Neighbor Distance (NND) analysis of ALFA-Nbs after RTX treatment shows non-CSR distributions for all shown NNDs. **e**, Optimizing the chain segment length in the flexible-chain model by calculating the sum of squared errors yields a chain segment length of 23 nm ± 2 nm. Monte-Carlo simulations (bottom) reveal an uncertainty of ± 2 nm. Centered vertical lines represent the means, and the error bars represent the standard deviations. Statistical significance was assessed using an unpaired t-test, with $p_{21,23}$=0.0043 and $p_{23,25}$=0.009. **f,** NND analysis of CD20 after OBZ treatment shows non-CSR distributions for 1st to 3rd NNDs. **g,** NND analysis of ALFA-Nb after OBZ treatment of CD20 shows non-CSR distribution for 1st NNDs only. **h**, C1q platform analysis for OBZ shows almost no platforms as most OBZ clusters are < 6 OBZ. **i,** Angle analysis for OBZ-CD20 clusters shows a 25°angle for ϕ (like in **Fig. 3**) and a random angle for θ. Created with the help of BioRender (https://BioRender.com/y76v9f4 and https://BioRender.com/5575210)

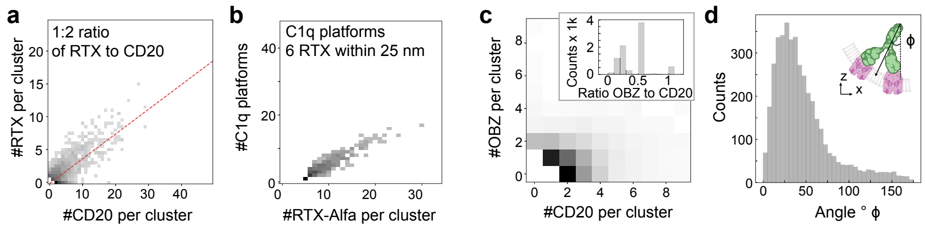

Supplementary Fig. 4 | Replicate for quantitative analysis of therapeutic antibody-CD20 complexes. a, Quantitative analysis of RTX-CD20 clusters reveals a linear relationship between the number of RTX molecules and CD20 dimers, suggesting that approximately one RTX molecule binds per CD20 dimer. **b**, There are multiple C1q-binding platforms within RTX-CD20 clusters, featuring at least 6 RTX within 25 nm. **c**, Quantitative analysis of OBZ-CD20 clusters reveals specific OBZ to CD20 stoichiometries, without a linear relationship between the number of OBZ and CD20 molecules. **d**, OBZ is bound to CD20 in a 25° angle over the xy-plane of the cell membrane. Created with the help of BioRender (https://BioRender.com/y76v9f4 and https://BioRender.com/5575210)

a DNA-PAINT and RESI NNDs after RTX-treatment

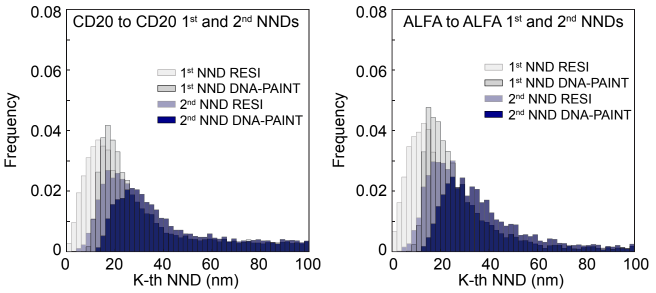

b DNA-PAINT and RESI NNDs after OBZ-treatment

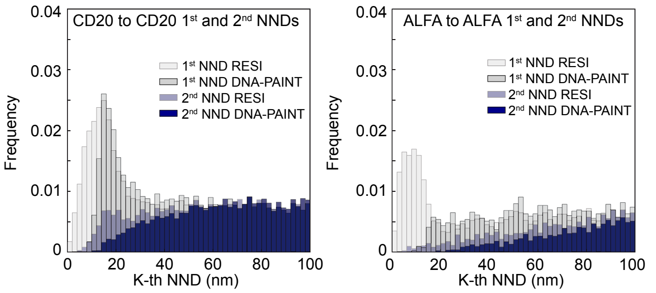

Supplementary Fig. 5 | NND analysis of DNA-PAINT and RESI data for RTX and OBZ treated cells. a, CD20 to CD20 and ALFA to ALFA NNDs obtained from analysis of DNA-PAINT and RESI data of RTX-ALFA-treated cells. Only first and second NNDs are displayed for clarity. The RESI NND histograms (high opacity) and the DNA-PAINT histograms (low opacity) are directly compared in one plot. After RTX treatment, DNA-PAINT NND data of CD20 shows first and second NND peaks (left). However, the resolution limit in DNA-PAINT limits the detection of sub-10 nm distances for the first NND. Only with RESI, these sub-10 nm distances are detected. Similar observations can be made for the ALFA to ALFA NNDs (right). **b**, CD20 to CD20 NNDs (left) and ALFA to ALFA NNDs (right) obtained from analysis of DNA-PAINT and RESI data of OBZ-ALFA-treated cells. Only first and second NNDs are displayed for clarity. The RESI NND histograms (high opacity) and the DNA-PAINT histograms (low opacity) are directly compared in one plot. After OBZ treatment, DNA-PAINT NND data of CD20 shows only a first NND peak. Only with RESI, a second NND peak, representing CD20 trimers and tetramers, can be detected. Sub-10 nm ALFA to ALFA NNDs for 2 ALFA-Nbs bound on one OBZ molecule (right) can only be detected with RESI but not with DNA-PAINT.

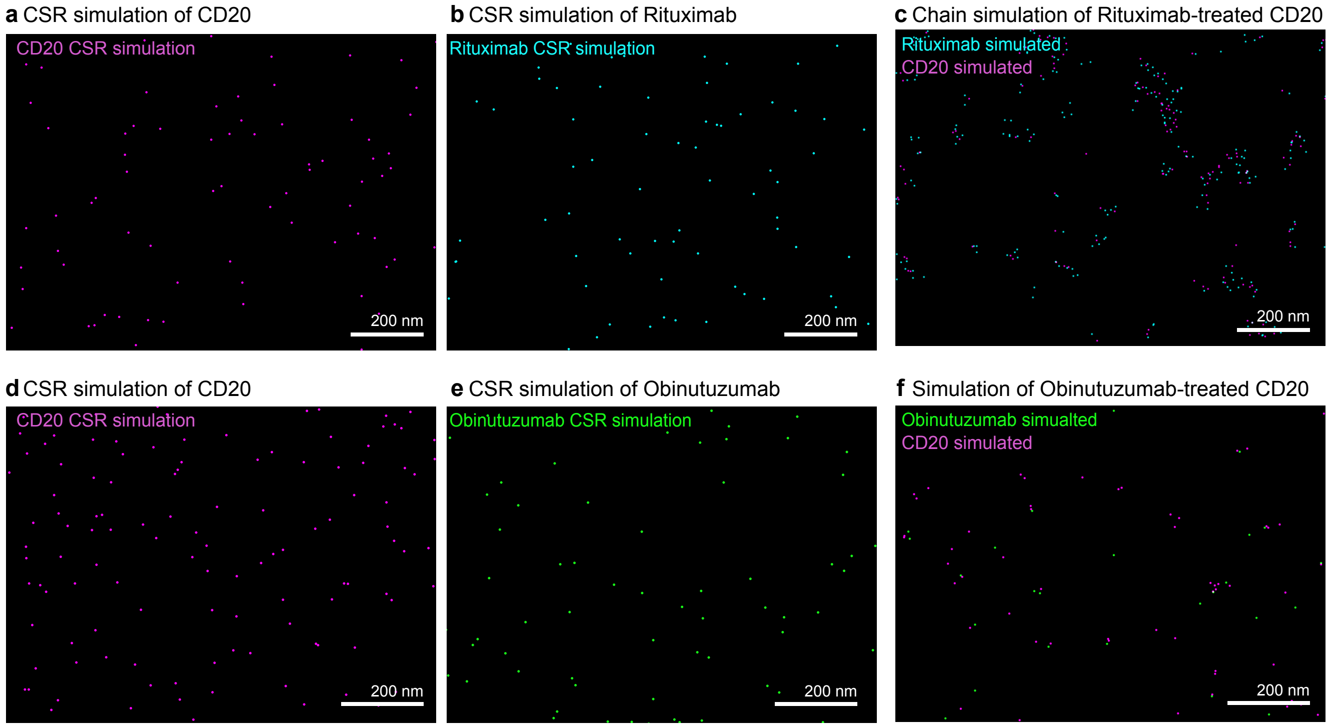

Supplementary Fig. 6 | Simulations of RTX-CD20 and OBZ-CD20 complexes for NND analysis. a, CSR monomer simulation of CD20 at the same density as measured in RESI data. **b**, CSR monomer simulation of ALFA labeling RTX at the same density as measured in RESI data. **c**, Chain simulation of CD20 and RTX with 23 nm chain segment length. **d**, CSR monomer simulation of CD20 at the same density as measured in RESI data. **e**, CSR monomer simulation of ALFA labeling OBZ at the same density as measured in RESI data. **f**, Simulation of OBZ bound to CD20 monomers, dimers, trimers and tetramers.

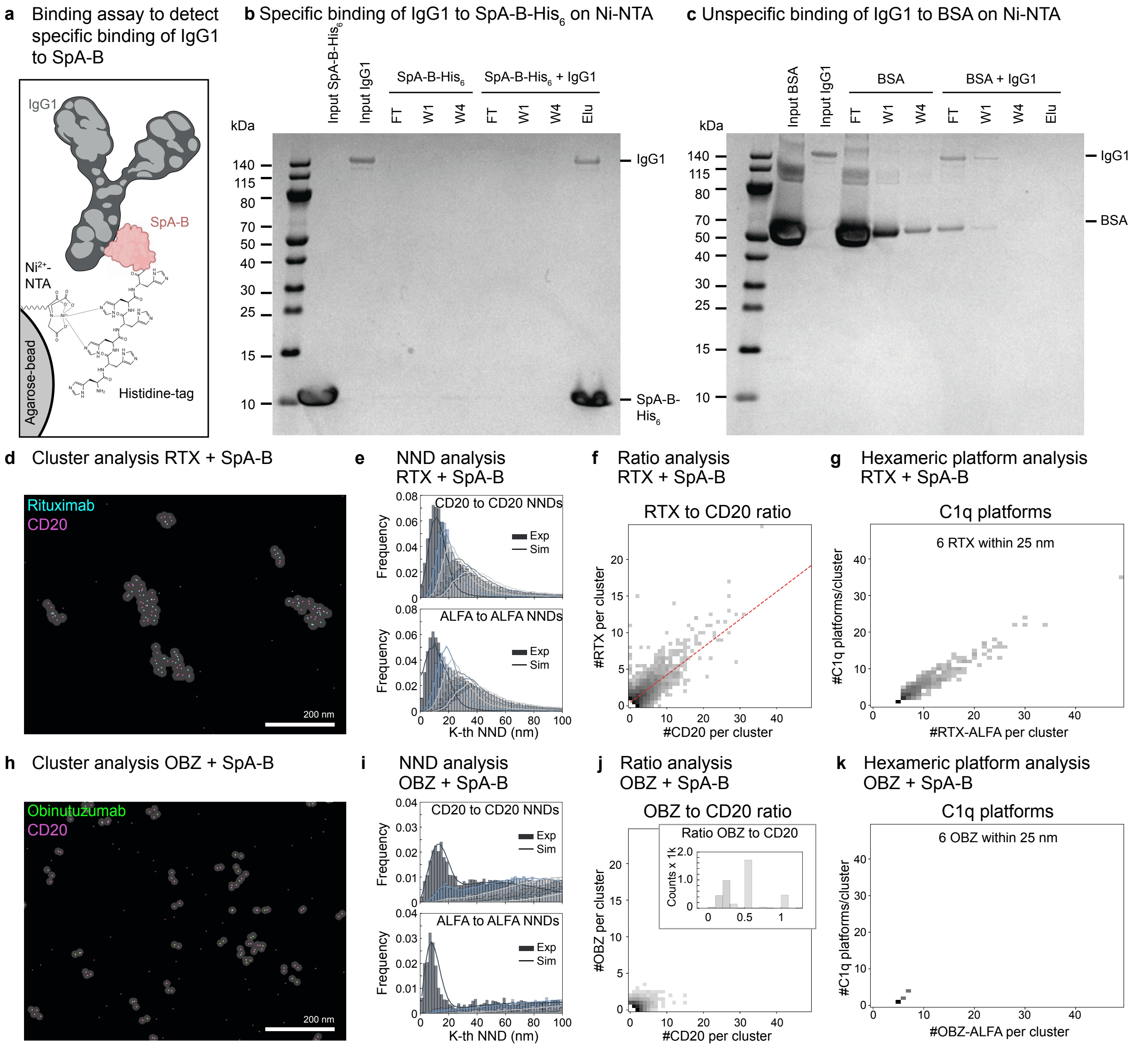

Supplementary Fig. 7 | CD20 and mAb organization upon Fc-Fc interaction blockade with SpA-B. a, Binding assay to test SpA-B binding to IgG1-Fc domain. SpA-B immobilized on Nickel-nitrilotriacetic acid (Ni-NTA) beads was incubated with IgG1. Created with the help of BioRender (https://BioRender.com/y76v9f4 and https://BioRender.com/5575210). **b**, SDS-PAGE analysis confirmed SpA-B binding to Ni-NTA beads, with no protein detected in flow-through (FT) and washes (W1, W4). After IgG1 incubation, neither SpA-B nor IgG1 were detected in FT or washes, indicating high-affinity binding. Elution confirmed specific binding of both proteins. **c**, SDS-PAGE of the negative control showed unspecific binding of BSA to Ni-NTA beads, as most protein appeared in FT and wash fractions. After IgG1 incubation, BSA and IgG1 were mostly detected in FT and washes, with no specific binding in elution. **d**, RESI imaging showed Rituximab (RTX)-CD20 clusters after SpA-B-blocked RTX treatment. DBSCAN analysis indicates similar higher-order structures to non-blocked samples. **e**, Nearest-Neighbor Distance (NND) analysis of blocked RTX-CD20 resembles non-blocked data, supported by flexible-chain simulations. **f**, Quantitative 2D cluster analysis indicates a linear relationship between RTX molecules and CD20 dimers. Two ALFA-Nbs corresponded to one RTX per cluster. When corrected for labeling efficiencies, this suggested ~1 RTX per CD20 dimer. g, Hexameric C1q platforms were equally detected in blocked and non-blocked RTX-CD20 samples. **h**, RESI imaging after SpA-B-blocked Obinutuzumab (OBZ) treatment showed dispersed clusters similar to non-blocked samples. **i**, NND analysis of blocked OBZ-CD20 identified peaks consistent with monomer to tetramer arrangements. NNDs of ALFA-Nbs labeling OBZ exluded higher-order clustering. **j**, Quantitative analysis revealed distinct OBZ-to-CD20 stoichiometries without linear relationships. Two ALFA-Nbs are displayed as one OBZ per cluster. **k**, Few C1q platforms were detected in OBZ-CD20 clusters, similar to non-blocked OBZ.

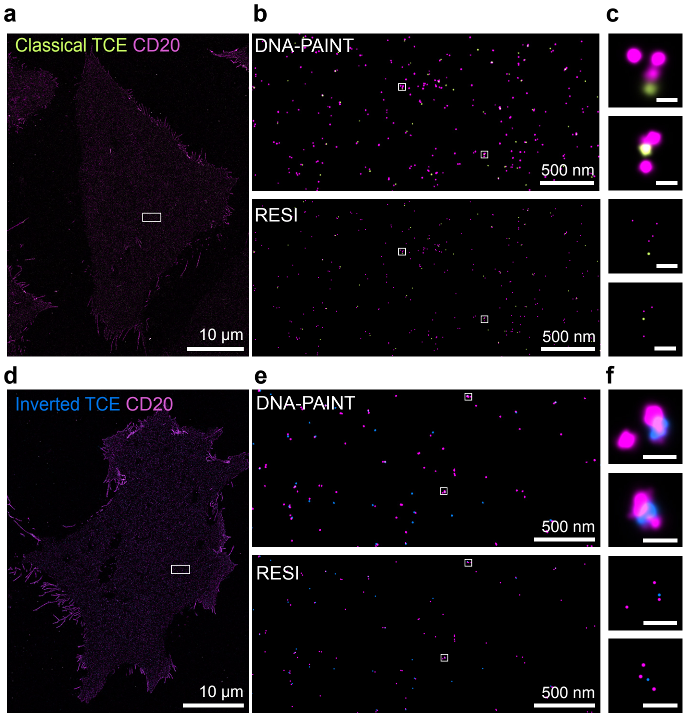

Supplementary Fig. 8 | Classical and inverted TCE bound to CD20. a, Whole cell DNA-PAINT image of c-TCE treated cells. **b,** Zoom in of c-TCE treated cells in DNA-PAINT (top) and RESI (bottom). **c,** Single c-TCE-CD20 complexes in DNA-PAINT and RESI (Scale bar: 20nm). **d,** Whole cell DNA-PAINT image of i-TCE treated cells. **e,** Zoom in of i-TCE treated cells in DNA-PAINT (top) and RESI (bottom). **f,** Single i-TCE-CD20 complexes in DNA-PAINT and RESI (Scale bar: 20nm).

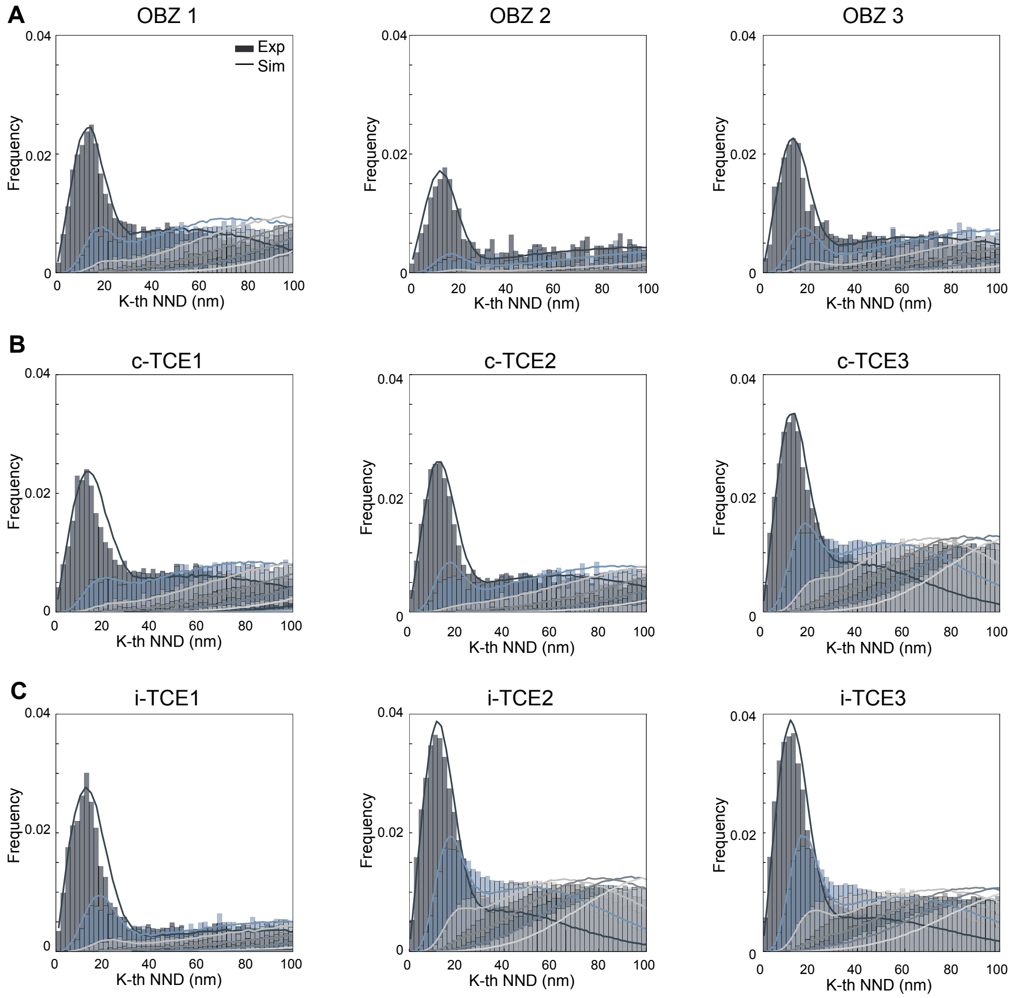

Supplementary Fig. 9 | Nearest-Neighbor Distance analysis of CD20 data upon mAb-treatment, depicting the low order oligomerization simulation results of Fig 4f. **a**, Three datasets of three individual OBZ-treated cells (Exp) with the simulation recapitulating the data (Sim). **b**, Three additional datasets of three individual c-TCE-treated cells (Exp) with the simulation recapitulating the data (Sim). **c**, Three additional datasets of three individual i-TCE-treated cells (Exp) with the simulation recapitulating the data (Sim).

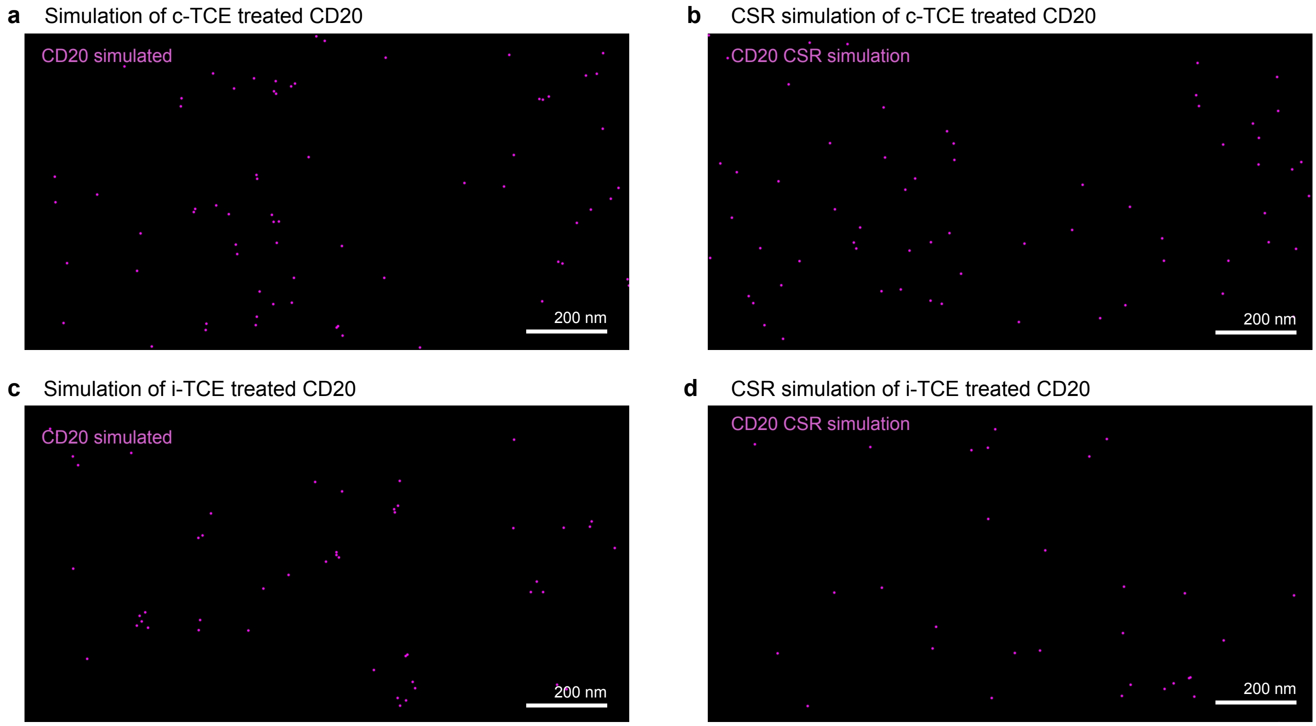

Supplementary Fig. 10 | Simulations of CD20 for NND analysis. a, Simulation of CD20 monomers, dimers, trimers and tetramers after c-TCE treatment. **b**, CSR monomer simulation of CD20 at the same density as measured for c-TCE treated CD20. **c**, Simulation of CD20 monomers, dimers, trimers and tetramers after i-TCE treatment. **d**, CSR monomer simulation of CD20 at the same density as measured for i-TCE treated CD20.

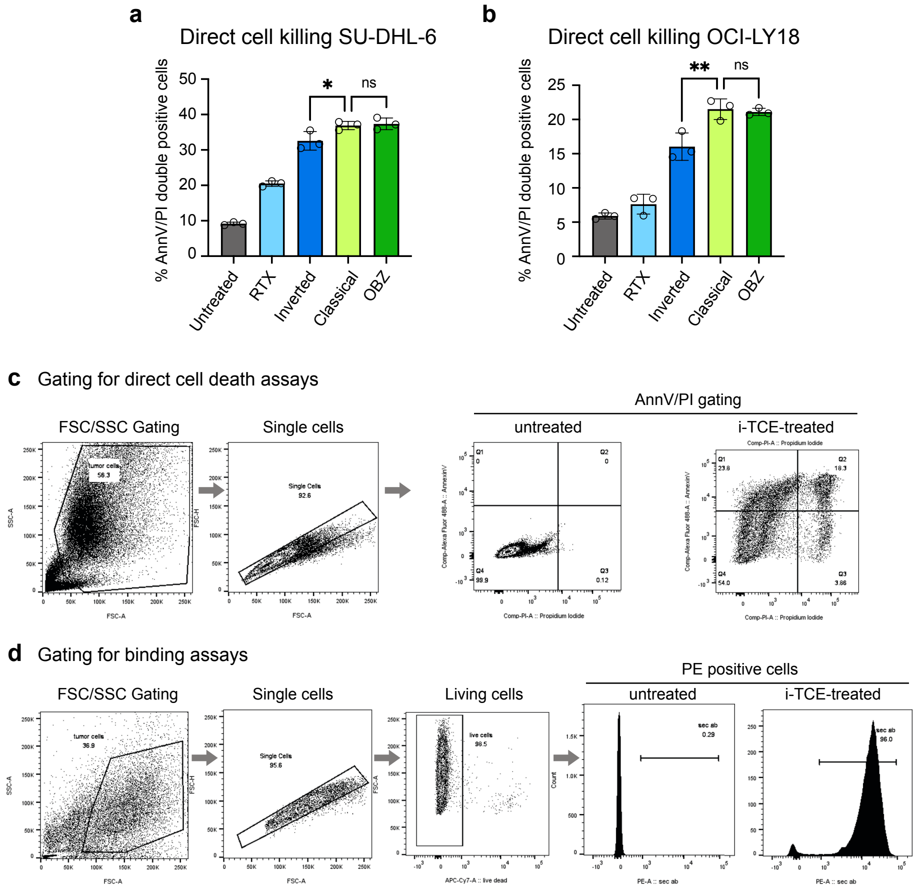

Supplementary Fig. 11 | Cell killing assays in CD20-positive cell lines. a, Direct cell killing FACS assay for untreated SU-DHL-6 cells, cells treated with Rituximab (RTX), classical TCE, inverted TCE and Obinutuzumab (OBZ). The results show a reduced killing efficiency for i-TCE vs c-TCE, trending toward values for Type I-RTX. The number of biological replicates is n=3. Statistical significance was tested using a one-way ANOVA, adjusting for multiple comparisons (p=0.0378). Source data are provided as a Source Data file. **b,** Direct cell killing FACS assay for untreated OCI-LY18 cells, cells treated with Rituximab (RTX), classical TCE, inverted TCE and Obinutuzumab (OBZ). The results show a reduced killing efficiency for i-TCE vs c-TCE, trending toward values for Type I-RTX. The number of biological replicates is n=3. Statistical significance was tested using a one-way ANOVA, adjusting for multiple comparisons (p=0.0037). Source data are provided as a Source Data file. **c,** Gating strategies for Fig. 4g. **d,** Gating strategies for Fig. 4h and Supplementary Fig. 11a,b.

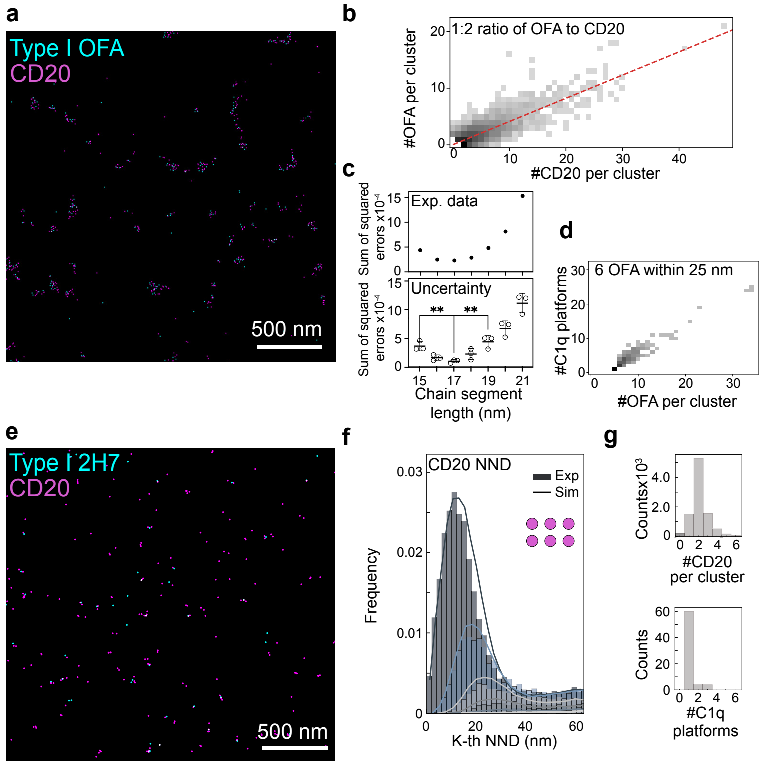

Supplementary Fig. 12 | Quantitative analysis of Type I therapeutic Antibody-CD20 complexes. a, RESI image of OFA-treated cells. **b,** Quantitative analysis of OFA-CD20 clusters reveals a linear relationship between the number of OFA molecules and CD20 dimers, suggesting that approximately one OFA molecule binds per CD20 dimer. **c,** Optimizing the chain segment length in the flexible-chain model by calculating the sum of squared errors yields a chain segment length of 17±2 nm. Monte-Carlo simulations (bottom) reveal an uncertainty of ±2 nm. Centered vertical lines represent the means, and the error bars represent the standard deviations. Statistical significance was assessed using an unpaired t-test, resulting in $p_{15,17}$=0.0063 and $p_{17,19}$=0.0064. **d,** Hexameric OFA platforms could facilitate complement component 1q (C1q) binding. **e,** RESI image of Type I 2H7-treated cells. **f,** NND analysis for CD20 complexes after 2H7-treatment shows linear chain-like hexameric CD20 complexes. **g,** The number of CD20 per cluster and the number of C1q platforms per cluster suggests the presence of CD20 hexamers after 2H7 treatment.

Supplementary Tables

Supplementary Table 1 | DNA-PAINT docking and imager sequences

Identity	Docking sequence	Imager sequence
5xR1	TCCTCCTCCTCCTCCTCCT	AGGAGGA-Cy3B
5xR2	ACCACCACCACCACCACCA	TGGTGGT-Cy3B
7xR3	CTCTCTCTCTCTCTCTCTC	GAGAGAG-Cy3B
7xR4	ACACACACACACACACACA	TGTGTGT-Cy3B
5xR5	CTTCTTCTTCTTCTTCTTC	GAAGAA-Cy3B
5xR6	AACAACAACAACAACAACAA	TGTTGTT-Cy3B
5xL1	left-handed TCCTCCTCCTCCTCCTCCT	left-handed AGGAGGA-Cy3B
7xL4	left-handed ACACACACACACACACACA	left-handed TGTGTGT-Cy3B

Supplementary Table 2 | DNA-conjugated Nanobodies

Name	Docking sequence	Stock Concentration (μM)
Anti-GFP-Nb-5xR1	TCCTCCTCCTCCTCCTCCT	5
Anti-GFP-Nb-5xR2	ACCACCACCACCACCACCA	5
Anti-GFP-Nb-7xR3	CTCTCTCTCTCTCTCTCTC	5
Anti-GFP-Nb-7xR4	ACACACACACACACACACA	5
Anti-GFP-Nb-7xL3	left-handed CTCTCTCTCTCTCTCTCTC	10
Anti-ALFA-Nb-5xR5	CTTCTTCTTCTTCTTCTTC	5
Anti-ALFA-Nb-5xR6	AACAACAACAACAACAACAA	5
Anti-ALFA-Nb-5xL1	left-handed TCCTCCTCCTCCTCCTCCT	10
Anti-ALFA-Nb-7xL4	left-handed ACACACACACACACACACA	10
Anti-human-IgG-Nb-7xR3 (2F2)	CTCTCTCTCTCTCTCTCTC	5
Anti-human-IgG-Nb-7xR3 (2H5)	CTCTCTCTCTCTCTCTCTC	5
Anti-mouse kappa light chain-Nb-7xR3	CTCTCTCTCTCTCTCTCTC	5

Supplementary Table 3 | Antibodies and Nanobodies

Name	Target	Format	Modification	Clone	Manufacturer	Cat.No.	Treatment/Staining	Concentration (nM)
Anti-GFP-Nb	GFP	Single domain antibody/ nanobody	C-terminal cysteine	1H1	NanoTag Biotechnologies	N0305	After PFA fixation	25
Anti-ALFA-Nb	ALFA	Single domain antibody/ nanobody	C-terminal cysteine	1G5	NanoTag Biotechnologies	N1505	After PFA fixation	25
Anti-Human IgG-Nb	Human IgG	Single domain antibody/ nanobody	C-terminal cysteine	2F3	NanoTag Biotechnologies	-	After PFA fixation	25
Anti-Human IgG-Nb	Human IgG	Single domain antibody/ nanobody	C-terminal cysteine	2H5	NanoTag Biotechnologies	-	After PFA fixation	25
Obinutuzumab	Human CD20	Human IgG1-defucosylated	ALFA-tag	GA101	Roche Glycart	-	Live	67
Rituximab	Human CD20	Human IgG1	ALFA-tag	Rituximab	Roche Glycart	-	Live	66.7
classical CD20-CD3-TCE	Human CD20, human CD3	Human IgG1, PG-LALA	-		Roche Glycart	-	Live	66.7
inverted CD20-CD3-TCE	Human CD20, human CD3	Human IgG1, PG-LALA	-		Roche Glycart	-	Live	6766.7
Ofatumumab	Human CD20	Human IgG1	-	2F2	Creative Biolabs	PABL-422	Live	66.7
Anti-CD20 (B1)	Human CD20	Mouse IgG2a, lambda	FITC	H299	Beckmann	6602381	Live	66.7
Ocrelizumab	Mouse IgG		-	2H7	eBioscience	14-0209-82	Live	66.7

Supplementary Table 4 | Buffer composition

Buffer name	Buffer composition
100× Trolox	100 mg Trolox, 430 µl 100 % Methanol, 345 µl 1M NaOH in 3.2 ml H_2O
40× PCA	154 mg PCA, 10 ml water and NaOH were mixed and pH was adjusted 9.0
100× PCD	9.3 mg PCD, 13.3 ml of buffer (100 mM Tris-HCl pH 8, 50 mM KCl, 1 mM EDTA, 50 % Glycerol)
Imaging buffer	1× PBS pH 7.4, 1 mM EDTA, 500 mM NaCl, 0.02% Tween; supplemented with 1× Trolox, 1× PCA and 1× PCD
Washing buffer	1× PBS pH 7.4, 0.02% Tween 20
Blocking buffer	1× PBS, 1 mM EDTA, 0.02% Tween-20, 0.05% NaN3, 2% BSA, 0.05 mg/ml sheared salmon sperm DNA
His-Incubation buffer	1xPBS pH 7.2, supplemented with 10 mM imidazole
His-Washing buffer	1xPBS pH 7.2, supplemented with 25 mM imidazole
His-Elution buffer	1xPBS pH 7.2, supplemented with 250 mM imidazole

Supplementary Table 5 | Imaging conditions and clustering parameters

Imager	Imager concentration [pM]	Integration time [ms] 2D	Integration time [ms] 3D	Frames	Clustering radius [nm]	Min number of locs
R1	500 pM	75	100	30000	6	15
R2	500 pM	75	100	30000	6	20
R3	500 pM	75	100	30000	6	15
R4	500 pM	75	100	30000	6	20
L3	500 pM	75	100	30000	6	15
R5	500 pM	100	100	30000	6	15
R6	500 pM	100	100	30000	6	15
L1	1000 pM	75	100	15000	6	15
L4	500 pM	75	100	30000	6	15
