# Supplementary material for: Resolving the structural basis of therapeutic antibody function in cancer immunotherapy with RESI
Source: Nat Commun. 2025 Jul 23;16:6768. doi: 10.1038/s41467-025-61893-w (PMC12284157; doi:10.1038/s41467-025-61893-w)
Supplement: Supplementary file 2 — Reporting Summary [file 41467_2025_61893_MOESM2_ESM.pdf]

nature portfolio

Corresponding author(s): Ralf Jungmann

Last updated by author(s): May 23, 2025

Reporting Summary

Nature Portfolio wishes to improve the reproducibility of the work that we publish. This form provides structure for consistency and transparency in reporting. For further information on Nature Portfolio policies, see our Editorial Policies and the Editorial Policy Checklist.

Statistics

For all statistical analyses, confirm that the following items are present in the figure legend, table legend, main text, or Methods section.

n/a	Confirmed	
☐	☒	The exact sample size (n) for each experimental group/condition, given as a discrete number and unit of measurement
☐	☒	A statement on whether measurements were taken from distinct samples or whether the same sample was measured repeatedly
☐	☒	The statistical test(s) used AND whether they are one- or two-sided<br>*Only common tests should be described solely by name; describe more complex techniques in the Methods section.*
☒	☐	A description of all covariates tested
☐	☒	A description of any assumptions or corrections, such as tests of normality and adjustment for multiple comparisons
☐	☒	A full description of the statistical parameters including central tendency (e.g. means) or other basic estimates (e.g. regression coefficient) AND variation (e.g. standard deviation) or associated estimates of uncertainty (e.g. confidence intervals)
☐	☒	For null hypothesis testing, the test statistic (e.g. F, t, r) with confidence intervals, effect sizes, degrees of freedom and P value noted<br>*Give P values as exact values whenever suitable.*
☒	☐	For Bayesian analysis, information on the choice of priors and Markov chain Monte Carlo settings
☒	☐	For hierarchical and complex designs, identification of the appropriate level for tests and full reporting of outcomes
☒	☐	Estimates of effect sizes (e.g. Cohen's d, Pearson's r), indicating how they were calculated

Our web collection on statistics for biologists contains articles on many of the points above.

Software and code

Policy information about availability of computer code

Data collection	The custom-built microscopy setup was controlled using µManager (Version 2.0.1) (Edelstein, A., Amodaj, N., Hoover, K., Vale, R. & Stuurman, N. Curr. Protoc. Mol. Biol. 14.20 (2010))
Data analysis	Raw image processing was performed using Picasso available via GitHub at https://github.com/jungmannlab/picasso

For manuscripts utilizing custom algorithms or software that are central to the research but not yet described in published literature, software must be made available to editors and reviewers. We strongly encourage code deposition in a community repository (e.g. GitHub). See the Nature Portfolio guidelines for submitting code & software for further information.

Data

Policy information about availability of data

All manuscripts must include a data availability statement. This statement should provide the following information, where applicable:

- Accession codes, unique identifiers, or web links for publicly available datasets
- A description of any restrictions on data availability
- For clinical datasets or third party data, please ensure that the statement adheres to our policy

RESI microscopy data obtained during this study are available from zenodo.

Research involving human participants, their data, or biological material

Policy information about studies with human participants or human data. See also policy information about sex, gender (identity/presentation), and sexual orientation and race, ethnicity and racism.

Reporting on sex and gender	n/a
Reporting on race, ethnicity, or other socially relevant groupings	n/a
Population characteristics	n/a
Recruitment	n/a
Ethics oversight	n/a

Note that full information on the approval of the study protocol must also be provided in the manuscript.

Field-specific reporting

Please select the one below that is the best fit for your research. If you are not sure, read the appropriate sections before making your selection.

☒ Life sciences ☐ Behavioural & social sciences ☐ Ecological, evolutionary & environmental sciences

For a reference copy of the document with all sections, see nature.com/documents/nr-reporting-summary-flat.pdf

Life sciences study design

All studies must disclose on these points even when the disclosure is negative.

Sample size	n/a
Data exclusions	No data was excluded
Replication	Each experiment was repeated independently at least N=3 times. All replications were successful.
Randomization	n/a
Blinding	n/a

Reporting for specific materials, systems and methods

We require information from authors about some types of materials, experimental systems and methods used in many studies. Here, indicate whether each material, system or method listed is relevant to your study. If you are not sure if a list item applies to your research, read the appropriate section before selecting a response.

Materials & experimental systems

n/a	Involved in the study	
☐	☒	Antibodies
☐	☒	Eukaryotic cell lines
☒	☐	Palaeontology and archaeology
☒	☐	Animals and other organisms
☒	☐	Clinical data
☒	☐	Dual use research of concern
☒	☐	Plants

Methods

n/a	Involved in the study	
☒	☐	ChIP-seq
☐	☒	Flow cytometry
☒	☐	MRI-based neuroimaging

Antibodies

Antibodies used	Rituximab (Roche Glycart), Obinutuzumab (Roche Glycart), Ofatumumab (2F2, Creative Biolabs), Ocrelizumab, Anti-CD20 (B1, H299, Beckmann), classical CD20-CD3-TCE(Roche Glycart), inverted CD20-CD3-TCE (Roche Glycart), anti-GFP-Nb (1H1, nanotag), anti-ALFA-Nb (1G5, nanotag), anti-human-Nb (2F3), anti-human Nb (2H5, nanotag)
Validation	The anti-CD20 antibodies were validated by Roche Glycart. The nanobodies were validated by nanotag.

Eukaryotic cell lines

Policy information about cell lines and Sex and Gender in Research

Cell line source(s)	CHO-K1 cell line (ATCC, CCL-61), Raji (ECACC #85011429), SU-DHL-6 (ATCC #CRL-2959), OCI-LY18 (DSMZ #ACC699)
Authentication	None of the cell lines used were authenticated.
Mycoplasma contamination	All cell lines have been tested negative for mycoplasma contamination
Commonly misidentified lines (See ICLAC register)	n/a

Plants

Seed stocks	n/a
Novel plant genotypes	n/a
Authentication	n/a

Flow Cytometry

Plots

Confirm that:

- [x] The axis labels state the marker and fluorochrome used (e.g. CD4-FITC).
- [x] The axis scales are clearly visible. Include numbers along axes only for bottom left plot of group (a 'group' is an analysis of identical markers).
- [x] All plots are contour plots with outliers or pseudocolor plots.
- [x] A numerical value for number of cells or percentage (with statistics) is provided.

Methodology

Sample preparation	Raji (ECACC #85011429) were cultivated in DMEM supplemented with 10 % FCS and 1 % Glutamax (Invitrogen/Gibco # 35050-038), SU-DHL-6 (ATCC #CRL-2959) and OCI-LY18 (DSMZ #ACC699) were cultivated in RPMI1640 supplemented with 10 % FCS and 1 % Glutamax. CD20 binding of obinutuzumab, rituximab, glofitamab and CD10 classical TCB was assessed on OCILY18, SUDHL6 and Raji B cell lines. The cell lines were resuspended at 1 x 106/ml in FACS Buffer and 100 µl/well (100000/ well) were seeded into 96-U-bottom plates. Antibody dilutions were prepared in FACS Buffer (200 nM down to 0.01 nM, 1:4 dilution steps). 25 µl/well of the pre-diluted antibodies or PBS were added after centrifugation to the cell pellets and incubated for 1 h at 4°C. Afterwards, cells were washed and incubated with a PE-labelled secondary antibody (PE-conjugated AffiniPure F(ab)'2 Fragment goat anti-human IgG Fcg Fragment specific (Jackson Immunoresearch Lab 109-116-170) in the presence of a live/dead marker (NIR) for 30 min at 4°C. Cells were washed twice and re-suspended in 150 µl/well FACS Buffer/PBS and measured using a BD FACS CantoII.
Instrument	BD FACS CantoII and BD FACSCantoTM II
Software	BD FACSCanto™ Clinical Software
Cell population abundance	Only CD20-positive cell lines were used (see above).
Gating strategy	Gating by FSC/SSC Gating => Single cells => Living cells => PE positive cells or FSC/SSC Gating => Single cells => AnnV/PI gating

- [x] Tick this box to confirm that a figure exemplifying the gating strategy is provided in the Supplementary Information.
